# Fetal agenesis of corpus callosum: chromosomal copy number abnormalities and postnatal follow-up

**DOI:** 10.1007/s11033-024-09821-x

**Published:** 2024-07-30

**Authors:** Meiying Cai, Na Lin, Meimei Fu, Yanting Que, Hailong Huang, Liangpu Xu

**Affiliations:** Medical Genetic Diagnosis and Therapy Center, Fujian Maternity and Child Health Hospital College of Clinical Medicine for Obstetrics & Gynecology and Pediatrics, Fujian Key Laboratory for Prenatal Diagnosis and Birth Defect, Fujian Clinical Research Center for Maternal-Fetal Medicine, Fujian Medical University, National Key Obstetric Clinical Specialty Construction Institution of China, Fuzhou, China

**Keywords:** Agenesis of the corpus callosum, Magnetic resonance imaging, Chromosomal abnormalities, Chromosomal microarray analysis, Postnatal follow-up

## Abstract

**Objective:**

Agenesis of the corpus callosum (ACC) is an anomaly that can occur in fetuses during pregnancy. However, there is currently no treatment for fetal ACC. Therefore, we conducted a retrospective analysis of obstetric outcomes of fetal ACC to explore the relationship between fetal ACC phenotypes and chromosomal copy number abnormalities.

**Methods and results:**

Amniotic fluid or umbilical cord blood were extracted from pregnant women with fetal ACC for karyotype analysis and chromosomal microarray analysis (CMA). Among the 48 fetuses with ACC, 22 (45.8%, 22/48) had isolated ACC, and 26 (54.2%, 26/48) had non-isolated ACC. Chromosomal abnormalities were detected via karyotype analysis in four cases. In addition to the four cases of pathogenic copy number variations (CNVs) detected using karyotype analysis, CMA revealed two cases of pathogenic CNVs with 17q12 microduplication and 16p12.2 microdeletion. The obstetric outcomes of 26 patients with non-isolated ACC were followed up, and 17 chose to terminate the pregnancy. In addition, seven of the nine cases with non-isolated ACC showed no obvious abnormality during postnatal follow-up, whereas only one case with normal CMA showed an abnormal phenotype at six months. Of the 22 patients with isolated ACC, six chose to terminate the pregnancy. Postnatal follow-up of 16 isolated ACC cases revealed only one with benign CNV, presenting with intellectual disability.

**Conclusion:**

Pregnant women with fetal ACC should be offered prenatal CMA, particularly non-isolated ACC. Patients with ACC should undergo prolonged postnatal follow-up, and appropriate intervention should be provided if necessary.

## Introduction

The development of the human brain, which begins in early pregnancy, is a gradual and highly complex process [1]. The corpus callosum is the white matter bundle that connects the left and right hemispheres of the brain. As the most crucial connecting pathway, the corpus callosum is mainly responsible for coordinating, transmitting, and integrating information between the two hemispheres of the brain [2]. The developmental process of the human corpus callosum is complex. Its basic form is established after 20 weeks of gestation. However, its development continues, and it grows longer, wider, and thicker postnatally [3]. The corpus callosum grows mainly from the anterior to the posterior, with the posterior genu forming first, followed by the body, splenium, anterior genu, and rostrum [4]. The bundle of fibers of the corpus callosum forms aboundary with the lateral ventricle and maintains its size and morphology.

Embryonic development of the corpus callosum may be affected by genetic and environmental factors, thus leading to agenesis of the corpus callosum (ACC) [5, 6]. ACC is a craniocerebral malformation with an incidence of 0.3–0.7% [7]. ACC is characterized by the enlargement of lateral ventricles, which are separated and deformed to varying degrees, as well as telencephalic hypoplasia, which causes enlargement and upward displacement of the third ventricle. Currently, fetal ACC etiology is attributed to genetic factors, prenatal infection, or toxic substances, among other causes. Approximately 30–45% of fetuses with abnormal corpus callosum development have a definite cause, 10% have chromosomal abnormalities, and 20–35% have identifiable genetic syndromes [8].

Owing to continuous progress in prenatal diagnosis technology, the accuracy of prenatal diagnosis of ACC has substantially improved, and an increasing number of researchers are paying attention to fetal ACC [3, 4]. Currently, fetal ACC is mainly diagnosed using ultrasonography and magnetic resonance imaging (MRI). Prenatal diagnosis of ACC after the second trimester is facilitated when using both ultrasonography and MRI [4]. In this study, 48 cases of fetal ACC (includes complete absence of ACC or absence of part of ACC) were retrospectively summarized, amniotic fluid or cord blood were extracted for karyotype analysis and chromosomal microarray analysis (CMA) detection, and pregnancy outcomes were followed to explore the relationship between chromosomal abnormalities and obstetric outcomes and to provide guidance for the clinical management of ACC.

## Materials and methods

### Patient recruitment

Forty-eight fetuses with ACC were diagnosed via MRI at Fujian Maternity and Child Health Hospital (a tertiary referral center), Fujian, China from January 2016 to October 2022. In this study, ACC included complete absence of ACC or absence of part of ACC. Among the 48 fetuses with ACC, 22 (45.8%, 22/48) had isolated ACC, and 26 (54.2%, 26/48) had non-isolated ACC. Isolated ACC is defined as ACC fetal phenotype, and non-isolated ACC includes other abnormal images besides ACC. This study included pregnant women aged between 21 and 45 years (average age 28.7 ± 3.6 years), and the gestational age ranged between 20.0 and 36.2 weeks (average 26 ± 4 weeks). All pregnant women signed informed consent statements prior to invasive diagnosis. Sampling involved parallel karyotype analysis and CMA. This study was approved by the Medical Ethics Committee of Fujian Maternity and Child Health Hospital, Fujian, China.

### Karyotype analysis

According to the different gestational ages, amniotic fluid (18–24 weeks) and umbilical cord blood (after 24 weeks) were extracted using ultrasonography for fetal karyotype analysis. Conventional culture, harvest, production, G-banding, scanning with an automatic scanner, and photography of fetal cells were conducted. G-banding karyotype analysis was performed according to the International Nomenclature System for Human Cytogenetics (ISCN2009). C and N bands were added if necessary. The karyotype count was increased if chromosomal abnormalities were identified.

### CMA

Genomic DNA was extracted from fetal cells. The Affymetrix CytoScan 750 K Array (Thermo Fisher Scientific, Waltham, MA, USA) was used for enzyme digestion, DNA digestion, purification, hybridization, washing, scanning, and analysis. All CNVs were analyzed at a resolution of 100 kb/50 markers. The PubMed database was used, referring to the Online Mendelian Inheritance in Man, Database of Genomic Variants, UCSC, Decipher, and other databases. The results of gene chip copy number variation (CNV) were analyzed and compared with clinical data and the American Society of Medical Genetics. According to the American Society of Medical Genetics and Genomics guidelines [9], CNV is divided into five levels: pathogenic CNV, likely pathogenic CNV, CNV with unclear clinical significance, likely benign CNV, and benign CNV. Our laboratory reported microdeletions or microduplications larger than 400 kb and not reported for variants of uncertain clinical significance CNVs with less than 1 Mb duplication and 500Kb deletion.

### Statistical analysis

SPSS 21.0 software (IBM, Armonk, NY, USA) was used for statistical analyses. Data are expressed in percentiles, and Fisher’s exact test was used for comparisons between groups. Statistical significance was set at *P* < 0.05.

### Obstetric and postnatal follow-up

All patients were followed up via telephone to determine the obstetric outcome, postnatal diagnosis, and treatment. The obstetrician carried out the telephone follow-up. The follow-up included physical growth and neurobehavioral development after birth. The evaluation of physical growth and neurobehavioral development was conducted by a pediatric neurologist. All adverse pregnancy outcomes, such as miscarriage, stillbirth, developmental abnormalities, neonatal and infant death, were followed up. Follow-up was performed until February 7, 2023. During postnatal follow-up, the youngest subjects were 1 year old, and the oldest were 5 years old. The median age at last evaluation was 2 years.

## Results

### Phenotypic characteristics of fetal ACC

Among the 48 fetuses with ACC, 22 (45.8%, 22/48) had isolated ACC, and 26 (54.2%, 26/48) had non-isolated ACC. Among the 26 cases of non-isolated ACC, 11 (42.3%, 11/26) had central nervous system abnormalities, including ventriculomegaly, choroid plexus cyst, and cerebellar vermis dysplasia, whereas 15 (57.69%, 15/26) had deformities outside the central nervous system, including seven cases of multiple system abnormalities, five cases of skeletal dysplasia, and two cases of ventricular septal defects (Table 1).

**Table 1:** Phenotypic characteristics of fetuses with non-isolated ACC

Phenotype	Number of cases(*n* = 26)
Associated with central nervous system abnormalities	
Ventriculomegaly	8
Choroid plexus cyst	2
Cerebellar vermis dysplasia	1
Associated with abnormalities outside the central nervous system	
Multiple systemabnormalities	7
Dysossification of the lateral sacrococcygeal vertebral arch	5
Ventricular septal defect	2
Separation of the double renal collecting system	1

ACC: agenesis of corpus callosum

### Karyotype analysis

Karyotype analysis was performed successfully on 48 fetuses with ACC, and chromosomal abnormalities were detected in four cases, with a detection rate of 8.3% (4/48). The four abnormal chromosomes included structural abnormality of chimera chromosome 13, large fragment deletion of chromosome 5, large fragment duplication of chromosome 17, and derived chromosome 6 abnormality (Table 2).

**Table 2:** Karyotype analysis of fetuses with ACC

Case	Karyotype analysis	CMA results	Phenotype	CNV classification	Obstetric outcome
1	46,XX, r(13)(p11q32)[61]/45,XX,-13[24]	arr[hg19]13q31.3q34(94,929,201 − 115,107,733)x1	ACC, FGR, Holoprosencephaly, Septum dysplasia	*P*	TP
2	46,XX, del(5)(p13)	arr[hg19]5p15.33p13.3(113,576 − 29,220,523)x1	ACC	*P*	TP
3	46,XY, dup(17)(p13q25)	arr[hg19]17p13.3p13.2(525,768,789)x1,17p13.2q25.3(5,768,958 − 80,004,050)x3 ∼ 4,17q25.3(80,008,255 − 81,041,823)x1	ACC, VSD	*P*	TP
4	46,XY, der(6)(pter→q27::p22.1→pter) 46,XY, der(6)(pter→q26::p21.3→pter)	arr[hg19]6p25.3p22.1(156,975 − 28,261,502)x3,6q27(170,384,298 − 170,914,297)x1	ACC, VSD, Ventriculomegaly, Thymus dysplasia	*P*	TP

CMA: chromosomal microarray analysis; CNV: copy number variation; FGR: fetal growth restriction; VSD: ventricular septal defect; P: pathogenic; TP: termination of pregnancy

### CMA

CMA was performed on 48 fetuses with ACC, and six pathogenic CNVs [detection rate: 12.5% (6/48)] and one benign CNV were detected (Table 3). In addition to the four pathogenic CNVs identified using karyotype analysis, CMA detected two additional pathogenic CNVs, 17q12 microduplication and 16p12.2 microdeletion. The17q12 microduplication contained a 1.5 Mb fragment, including the *LHX1* (601,999) and *HNF1B* (189,907) genes. The 16p12.2 microdeletion contained a 0.69 Mb fragment, including the *OTOA* (607,038), *UQCRC2* (191,329), *EEF2K* (606,968), *POLR3E* (617,815), and *CDR2* (117,340) genes. Parental origin verification using CMA showed that the 17q12 microduplication was inherited from the mother, whereas the parents of the fetus with the 16p12.2 microdeletion refused parental origin verification.

**Table 3:** CMA results in fetuses with ACC

Case	CMA results	Size (Mb)	Phenotype	CNV classification	Inheritance	Obstetric outcome
1	arr[hg19]17q12(34,822,465 − 36,378,678)x3	1.5	ACC, Ventriculomegaly	*P*	Maternal	Language retardation
2	arr[hg19]16p12.2(21,740,200 − 22,442,007)x1	0.69	ACC, BPD and HC < M-2SD, Cerebellar dysplasia	*P*	Refuse	TP
3	arr[hg19]5q35.3(179,194,643 − 179,767,135)x3	0.56	ACC	LB	Maternal	Developmental delay

BPD: biparietal diameter; HC: head circumference; LB: likely benign; P: pathogenic; SD: standard deviation

### Comparison of pathogenic CNV rate between the isolated and non-isolated fetal ACC groups

Pathogenic CNVs were detected in only one of 22 (4.5%) isolated ACC cases and five of 26 (19.2%) non-isolated ACC cases. Although the rate of pathogenic CNVs in the non-isolated ACC group was higher than that in the isolated ACC group, the difference between the two groups was not statistically significant (*P* = 0.199) (Table 4).

**Table 4:** Differences in pathogenic CNVs between the two groups

Classification	Number of fetuses	Pathogenic CNV
Isolated ACCgroup	22	1(4.5%)
Non-isolated ACCgroup	26	5(19.2%)

ACC: agenesis ofthe corpus callosum; CNV: copy number variation

### Obstetric outcomes and postnatal follow-up

All 48 fetuses with ACC were successfully followed up. Of the 26 cases with non-isolated ACC, 17 patients (four of which harbored pathogenic CNVs) chose to terminate the pregnancy, whereas the other nine chose to continue the pregnancy. Among these nine non-isolated ACC cases, seven had no apparent abnormalities during postnatal follow-up. In contrast, only one case among the non-isolated ACC exhibiting the “small for gestational age” intrauterine phenotype had low height, weight, and developmental delay at six months after birth. Another non-isolated ACC case with ventriculomegaly and maternal 17q12 microduplication exhibited language delay at two years after birth. Of the 22 isolated ACC cases, six patients (including one with pathogenic deletion of chromosome 5) chose to terminate the pregnancy, whereas the other 16 chose to continue the pregnancy. Postnatal follow-up of the 16 isolated ACC cases revealed only one case of likely benign CNV with intellectual disability, whereas no abnormality was observed in the other 15 cases (Table 5).

**Table 5:** Pregnancy outcomes and postnatal follow-up of 48 fetuses with ACC

Phenotype	Pregnancy	outcome and postnatal	follow-up	Total
	TP	Postnatal normality	Postnatal abnormality	
Isolated ACC	6	15	1	22
Non-isolated ACC	17	7	2	26

ACC: agenesis ofthe corpus callosum; CNV: copy number variation; TP: termination of pregnancy

## Discussion

Common causes of ACC include chromosomal abnormalities, metabolic diseases in pregnant women, intrauterine infections, and exposure to teratogenic substances [10]. Approximately 10–17% of ACC cases have chromosomal abnormalities, with aneuploidy being the most common (Trisomy 18 and trisomy 13 are the most common) [11]. In this study, karyotype analysis revealed four cases of chromosomal abnormalities, with an abnormality rate of 8.3%. However, all chromosomal abnormalities were unbalanced.

The two pathogenic CNVs detected via CMA were 17q12 microduplication and 16p12.2 microdeletion, with the former showing autosomal dominant inheritance. In the present case of fetal 17q12 microduplication, the mother showed a normal phenotype and normal brain MRI. The 17q12 microduplication contained a 1.5 Mb fragment, including the *LHX1* and *HNF1B* genes. The clinical phenotypes of 17q12 microduplications vary substantially and may manifest as developmental delay/intellectual disability, behavioral problems, microcephaly, epilepsy, ACC, mild facial abnormalities, esophageal atresia, and urinary system abnormalities, while some patients may have no apparent clinical abnormalities [12]. The fetus with 17q12 microduplication in this study exhibited ACC, consistent with the afore mentioned reports. The 16p12.2 microdeletion is a susceptible site for neurodevelopment, with a penetrance of approximately 12.3%. Clinical phenotypes vary widely and maymanifest as developmental delay, mild to moderate intellectual impairment, language delay, mental and behavioral abnormalities, microcephaly, ACC, congenital heart defects, sleep disorders, and epilepsy [13, 14]. The intrauterine ultrasound phenotypes of the fetus in this study included bone abnormalities, cerebellar dysplasia, and ACC, also consistent with the afore mentioned literature reports. Previous studies have shown that 8p23 microduplication, as well as 1p36 and 1q42-43.6 microdeletions are all associated with ACC [15–17]. 8p23 microduplication, as well as 1p36 and 1q42-43.6 microdeletions were not found in this study. The molecular mechanism of 8p23 microduplication, 1p36 and 1q42-43.6 microdeletions leading to ACC needs to be further studied in the future.

Current studies have differing views on ACC prognosis [18, 19]. Consequently, prenatal counseling in ACC is highly challenging. The identification of abnormal CNVs using CMA is common in cases of non-isolated ACC. The poor prognosis of non-isolated ACC mainly depends on the type of concomitant malformation, particularly the prognosis of severe neurological malformations [19]. In this study, 17 of the 26 pregnancies with non-isolated ACC were terminated. During postnatal follow-up, seven cases of non-isolated ACC had no apparent clinical abnormal phenotypes (seven cases were within the normal range despite the malformations). Patients with isolated ACC range from completely asymptomatic to showing mild motor, language, and learning disorders, and even severe neurological and intellectual disabilities, epilepsy, and autism, if the etiology is unknown [20–22]. Mangione et al. reported that up to 70% of children with a prenatal diagnosis of isolated ACC developed normally [23]. In addition, des Portes et al. indicated that intellectual ability is normal in approximately 2/3 and borderline in just over 1/4 of patients with isolated ACC [24]. Sotiriadis et al. reported proportions of normal neurodevelopment and severe disability of 75.4% and 11.6% in children with isolated ACC, respectively [25]. In this study, 16 isolated ACC cases were followed up after birth, and no abnormalities were observed, except for one case of intellectual disability. However, long-term follow-up is required, and corresponding intelligence tests should be conducted regularly to understand the developmental status of the nervous system.

This study had some limitations. First, ACC is a craniocerebral malformation disease; therefore, the sample size was small, which may affect the conclusion; thus, further investigation of a larger group is required. Second, the latest whole genome sequencing has not been applied to ACC fetuses with normal CMA results [26–30]; this may have resulted in misdiagnoses of gene pathogenicity in this study. Third, the follow-up time for patients with ACC after birth was relatively short. Some disorders, such as attention deficit hyperactivity disorder, autism, and executive dysfunction, can only be detected when patients with ACC have reached a certain stage of development, and they are commonly diagnosed after the age of 10 years [8, 31]. Mild to moderate intellectual development disorders are only detectable at the age of 6 years old. Therefore, the time of disease occurrence should be considered in future studies, and the time limit of follow-up for patients with ACC should be extended.

## Conclusion

In conclusion, pregnant women should be offered prenatal fetal CMA examination when clinically diagnosed with ACC, particularly non-isolated ACC. In genetic counseling, the fetus with ACC should be comprehensively evaluated, and a final decision should be made regarding its future. Prenatal exome sequencing should be performed, and follow-up should be strengthened for fetuses with ACC with normal CMA. Appropriate intervention should be provided when necessary, and a long-term follow-up observation system should be established to understand the ACC prognosis and provide a framework for prenatal genetic counseling.

## Data Availability

Raw data for dataset are not publicly available to preserve individuals’ privacy under the Chinese General Data Protection Regulation.
